# Characterization of B- and T-Cell Compartment and B-Cell Related Factors Belonging to the TNF/TNFR Superfamily in Patients With Clinically Active Systemic Lupus Erythematosus: Baseline BAFF Serum Levels Are the Strongest Predictor of Response to Belimumab after Twelve Months of Therapy

**DOI:** 10.3389/fphar.2021.666971

**Published:** 2021-05-21

**Authors:** Silvia Piantoni, Francesca Regola, Stefania Masneri, Michele Merletti, Torsten Lowin, Paolo Airò, Angela Tincani, Franco Franceschini, Laura Andreoli, Georg Pongratz

**Affiliations:** ^1^ Rheumatology and Clinical Immunology Unit, ASST Spedali Civili of Brescia, Department of Clinical and Experimental Sciences, University of Brescia, Brescia, Italy; ^2^ Department of Rheumatology and Hiller Research Center for Rheumatology, University Hospital Düsseldorf, Düsseldorf, Germany

**Keywords:** systemic lupus erythematosus, belimumab, biomarkers, TNF/TNFR superfamily-related factors, adaptive immunity

## Abstract

**Background:** Patients with systemic lupus erythematosus (SLE) show increased serum levels of tumor necrosis factor (TNF)/TNF receptor (R) superfamily member, e.g. BAFF (B lymphocyte stimulator). Belimumab, a monoclonal antibody against soluble BAFF, is used for treatment of SLE. Although B cells are the main target, a BAFF-dependent T-cell activation pathway also plays a role. High levels of anti-DNA antibodies and low complement at baseline are known predictors of response to Belimumab.

**Objectives:** To explore the association of circulating lymphocytes and serum levels of B- cell related TNF/TNFR superfamily members with response to Belimumab in SLE patients.

**Methods:** Twenty-one SLE patients received Belimumab. Clinical evaluation and laboratory tests were performed at baseline, at 6 and 12 months. TNF super-family members (BAFF, APRIL, sBCMA, sCD40L, sTACI, TWEAK) were tested by high-sensitivity ELISA in all patients, and lymphocyte immunophenotyping was performed by flow cytometry in ten subjects. SLE-disease activity was assessed by SLEDAI-2K score. Linear regression modeling was used to investigate parameters influencing SLEDAI-2K and anti-dsDNA antibody titers over time and for predictive models.

**Results:** Clinical improvement was observed in all patients. A global reduction of circulating B cells, especially naïve, was detected, without variation in the T-cell compartment. All TNF family members decreased, whereas APRIL remained constant. The increase in serum levels of C3 (*p* = 0.0004) and sTACI (*p* = 0.0285) was associated with a decrease of SLEDAI-2K. The increase of C4 (*p* = 0.027) and sBCMA (*p* = 0.0015) and the increase of CD8^+^ T cells (*p* = 0.0160) were associated with a decrease, whereas an increase of sCD40L in serum (*p* = 0.0018) and increased number of CD4^+^ T cells (*p* = 0.0029) were associated with an increase, in anti-dsDNA antibody titers, respectively. Using stepwise forward inclusion, the minimal model to predict SLEDAI-2K response at 12 months included BAFF (*p* = 3.0*e* − 07) and SLEDAI-2K (*p* = 7.0*e* − 04) at baseline. Baseline APRIL levels also showed an association, although the overall model fit was weaker.

**Conclusion:** In our real-life cohort, baseline serum levels of BAFF were the best predictor of response to Belimumab, confirming post-hoc results of the BLISS study and suggesting the utility of this particular biomarker for the identification of patients who are more likely to respond.

## Introduction

An imbalance of B- and T-cell activity and differentiation was described as a crucial pathogenetic event in systemic lupus erythematosus (SLE) (Nagy et al., 2005). The pivotal role of autoantibodies was accepted as one of the main events in SLE and all factors which are involved in their development were studied as potential triggers of the disease (Nagy et al., 2005). BAFF (B-cell activating factor), also known as BLyS (B lymphocyte stimulator), is a member of Tumor necrosis factor/Tumor necrosis factor receptor (TNFS/TNFR) superfamily, also including APRIL (a proliferation-inducing ligand), their common receptors TACI (transmembrane activator and calcium-modulator and cyclophilin ligand interactor) and BCMA (B cell maturation antigen), CD40 ligand (CD40L) and TWEAK (TNF-related weak inducer of apoptosis). These factors, which show a high degree of structural homology with TNF, are described to be widely involved in the pathogenesis of SLE and in other systemic autoimmune diseases and they are newly identified as possible target of therapies (Ware, 2013).

Experimental data on SLE mouse models showed that BAFF and APRIL act in a concert to support humoral memory (Samy et al., 2017). BAFF is crucial for the development of self-reactive B cells from the transitional stage, which are more dependent on BAFF for their survival than memory B cells (Lesley et al., 2004; Samy et al., 2017). APRIL seems to act at a later stage, promoting the establishment of long-lived plasma cells (Belnoue et al., 2008). Both are involved in B-cell activation and class-switch recombination. BAFF and APRIL bind to TACI and BCMA, while BAFF additionally binds to a third receptor, BAFF-R. These receptors are expressed on the membrane and shed by B lineage cells during their differentiation (Darce et al., 2007) and BAFF-R and BCMA are described to be expressed also by T cells (Ng et al., 2004). CD40L ligand is the molecule which binds CD40, a stimulatory receptor expressed on dendritic cells, macrophages and B cells. It is crucial in IgG immunoglobulin class switching (Peters et al., 2009). CD40L is also fundamental as co-stimulatory molecule displayed on the membrane of T cells during the early phase of activation (Grewal and Flavell, 1996). CD40/CD40L blockade has been successful in preventing or stabilizing SLE nephritis in murine models (Kalled et al., 1998). TWEAK, produced by a large amount of myeloid and immune cells, is a factor acting primarily on tissue cells. In fact, its receptor, the fibroblast growth factor-inducible 14, is highly expressed on non-hematopoietic cells and up-regulated by injury-associated factors (Burkly, 2014). Dysfunction of TWEAK or its receptor has been described in the pathogenesis of lupus nephritis (Zhao et al., 2007).

Several drugs blocking the above factors were tested in clinical trials for their use in selected SLE patients. Only Belimumab, a fully humanized monoclonal IgG1λ antibody neutralizing soluble BAFF, has been approved for treatment of clinically active SLE (Navarra et al., 2011). A pooled subgroup analyses of Belimumab trials over 52 weeks of treatment (BLISS-52) demonstrated a greater therapeutic benefit in patients with increased disease activity at baseline, as measured by Safety of Estrogens in Lupus Erythematosus National assessment-Systemic Lupus Erythematosus Disease Activity Index (SELENA-SLEDAI) (Furie et al., 2011), anti-double strand (ds) DNA positivity, low complement or corticosteroid treatment (Navarra et al., 2011). As suggested by the post-hoc analyses of the Belimumab trials (BLISS-52 and BLISS-76) (Zhao et al., 2007; Burkly, 2014), baseline BAFF levels were proposed to be potentially useful in identifying SLE patients in which Belimumab might be expected to be more successful (Roth et al., 2016).

The objective of the present study is to characterize circulating peripheral B and T lymphocytes together with the evaluation of soluble B-cell related factors belonging to the TNF/TNFR superfamily in a real-life cohort of clinically active SLE patients treated with Belimumab, in order to explore the potential role of these pathogenetic factors as predictors of response to therapy.

## Patients and Methods

### Patients

Twenty-one consecutive patients with SLE, classified according to the revised American College of Rheumatology (ACR) criteria (Hochberg, 1997), and treated with Belimumab according to common clinical practice, were enrolled in this study. Written informed consent was obtained from all patients. Their main clinical, laboratory and demographic features, obtained from clinical records, are presented in Table 1. 76% of patients took immunosuppressants: seven were on treatment with mycophenolate mofetil at the median dose (10th–90th percentile) of 2 (1.6–2) g/die, four with methotrexate at 15 (12–15) mg/week, four with azathioprine at 100 (75–100) mg/die, one with cyclosporine at 250 mg/die. SLE Disease Activity Index 2000 (SLEDAI-2K) score was used to determine disease activity (Romero-Diaz et al., 2011).

**TABLE 1:** Demographic, clinical and laboratory features of SLE patients at baseline.

	Total patients (*n* = 21)	Patients with lymphocyte immuno-phenotyping (*n* = 10)	*p*
Demographic features			
Women (*n*, %)	19 (90)	8 (80)	0.5773
Age, years	41 (31–58)	39 (33–45)	0.5671
Disease duration, years	10 (2–23)	12 (7–20)	0.4443
SLE Manifestations			
Cutaneous manifestations (malar rash and/or discoid rash, oral ulcers)	18 (86)	9 (90)	1.0000
Articular involvement (arthritis/Jaccoud’s arthropathy)	19 (90)	9 (90)	1.0000
Renal involvement	11 (52)	5 (50)	1.0000
Hematological involvement	11 (52)	4 (40)	0.7043
NPSLE	5 (24)	3 (30)	1.0000
Serositis (pulmonary/pericardic effusion)	4 (19)	3 (30)	0.6518
Antiphospholipid syndrome	3 (14)	2 (20)	1.0000
SLEDAI 2K-score	6 (4–10)	6 (4–9)	0.5054
Laboratory Parameters			
Blood count of leukocytes (X10^9^/L) (*nv* = 4–9)	6 (2–8)	6 (5–8)	0.3859
Serum levels of C3 (mg/dl) (*nv* = 80–160)	72 (52–97)	68 (53–90)	0.6266
Serum levels of C4 (mg/dl) (*nv* = 10–40)	9 (6–18)	10 (7–17)	0.5965
Serum levels of anti-dsDNA (UI/ml) (*nv* < 7)	26 (7–128)	37 (8–289)	0.6051
aCL positivity (IgM and/or IgG) (*n*, %)	6 (29)	2 (20)	1.0000
Anti-b2GPI positivity (IgM and/or IgG) (*n*, %)	6 (29)	2 (20)	1.0000
LA positivity (*n*, %)	6 (29)	3 (30)	1.0000
Treatment^a^			
Dosage of prednisone (mg/day)	9 (5–23)	8 (5–23)	0.6113
Use of hydroxychloroquine at 5 mg/kg/day (*n*, %)	15 (71)	7 (70)	1.0000
Use of immunosuppressant drugs (*n*, %)	16 (76)	7 (70)	0.4648

Data are expressed as median (10th–90th percentile), if not otherwise specified. NPSLE, neuropsychiatric systemic lupus erythematosus; SLEDAI-2K score, systemic lupus erythematosus disease activity index 2000; C3 and C4: complement factor 3 and 4; Anti-dsDNA, anti-double-stranded DNA autoantibody; aCL, anti-cardiolipin antibodies; Ig, immunoglobulin; Anti-b2GPI, antibeta2-glycoprotein I antibodies; LA, lupus anticoagulant; nv, normal values.

a Seven patients were on treatment with mycophenolate mofetil, four patients with methotrexate, four with azathioprine, one with cyclosporine.

The study was approved by the local institutional ethics committee (approval number 2793) and conducted in accordance with the Declaration of Helsinki.

### Laboratory Parameters

**FIGURE 1:**
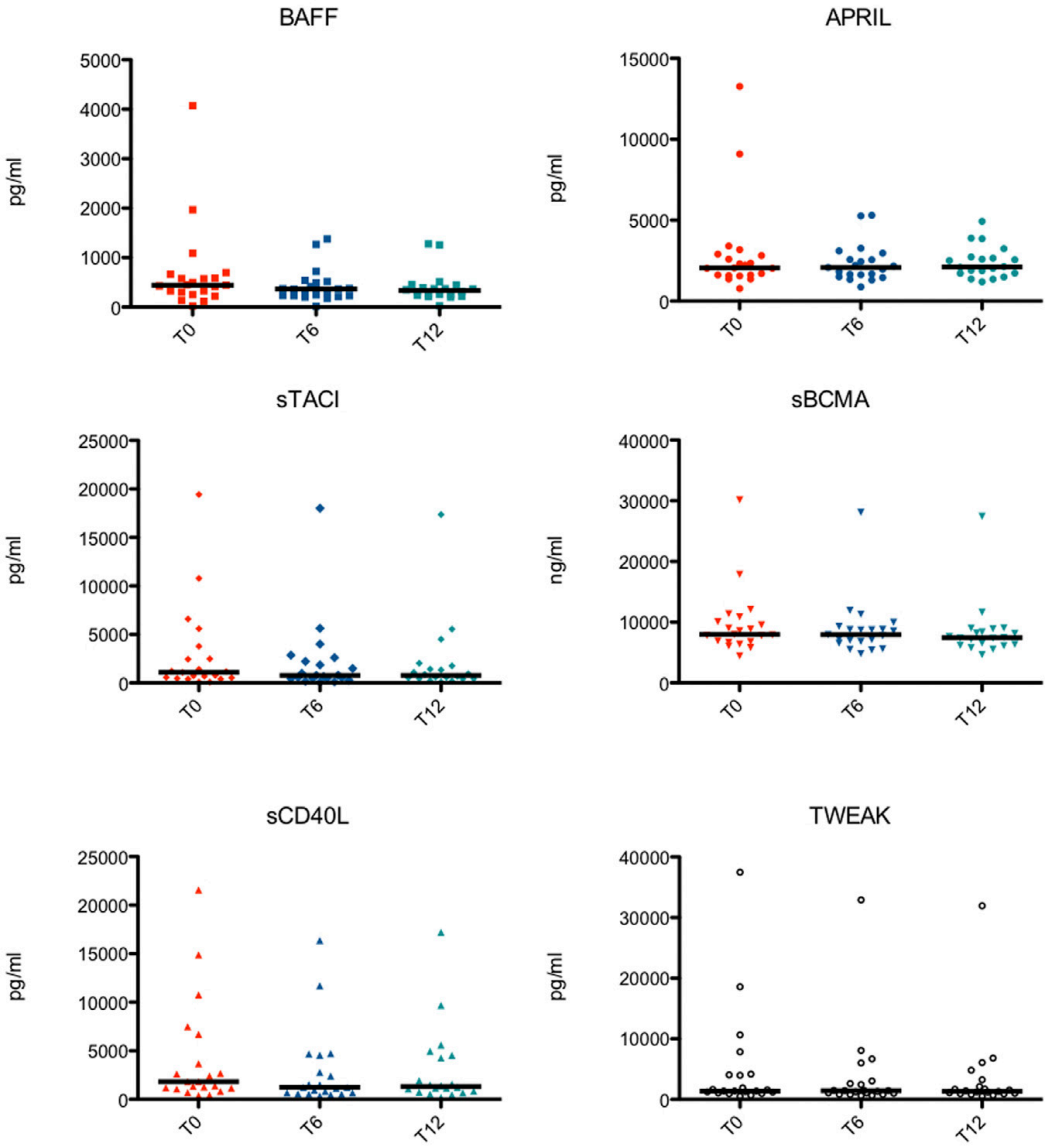
Serological levels of the TNF Superfamily members at different time points in each subject. BAFF, B cell activating factor; APRIL, a proliferation-inducing ligand; sTACI, soluble transmembrane activator and calcium-modulator and cyclophilin ligand interactor; sBCMA, soluble B cell maturation antigen; sCD40L, soluble CD40 ligand; TWEAK, TNF-related weak inducer of apoptosis.

Peripheral blood samples of 21 patients were obtained at the start of the study (T0) and every six months of treatment (T6 and T12). Only one dosage of the TNF/TNFR superfamily members at T12 of one patient was missing (Figure 1).

Anti-dsDNA autoantibodies were determined by FARR assay (Kodak Clinical Diagnostics, Amersham, United Kingdom) and C3 and C4 levels by nephelometry (Siemens Healthcare, Deerfield, IL, United States).

BAFF, APRIL, sTACI, sBCMA, sCD40L, and TWEAK levels were measured by respective commercially available ELISAs (human Duo Set; R&D Systems, Inc., Minneapolis, MN, United States), according to manufacturer’s guidelines.

### Flow Cytometry

In the first ten enrolled subjects, lymphocyte immunophenotyping was performed by flow cytometry.

One hundred microliters of whole blood were stained for 30 min at 4°C using monoclonal antibodies conjugated with fluorochromes (Beckman Coulter Inc., Fullerton, CA, United States) to identify B and T-cell surface markers by flow cytometry (Cytomics NAVIOS, Beckman Coulter), as previously described (Piantoni et al., 2018; Regola et al., 2019). Absolute cell count was determined by single platform analysis using Flow-Count beads (Beckman Coulter), according to manufacturer’s guidelines.

### Statistical Analysis

Data are expressed as median (10th–90th percentile). Comparisons between groups were made with Mann-Whitney test or Wilcoxon signed-rank test, when appropriated. Spearman rank test was used to evaluate the correlations between quantitative variables. Chi-square test or Fisher’s exact test were applied for comparison between qualitative variables. Robust Mixed linear regression modeling (R package: robustlmm Koller (2016)) was used to investigate parameters influencing SLEDAI-2K and anti-ds DNA antibody titers over time. Adjustment for intra-individual effects was done by including patient ID as a random intercept in the linear regression model. Absolute numbers of CD19^+^, CD4^+^, CD8^+^, CD4^+^CD28^−^ and T regulatory cells as well as leukocytes and serum levels of C3, C4, BAFF, APRIL, sTACI, sBCMA, sCD40L, TWEAK and anti-dsDNA antibodies were included in the initial model. Model selection was done by stepwise backwards exclusion. The time variable was kept in all models. Predictive linear models were built by stepwise forward inclusion using the same basic model structure. Statistical analysis was performed by using the software package GraphPad Prism six software and R software package version 4.0.3. [R Core Team (2020), R Foundation for Statistical Computing, Vienna, Austria]. *p*-values (*p*) ≤ 0.050 were considered as statistically significant.

## Results

### Clinical and Laboratory Features of Systemic Lupus Erythematosus Patients Treated With Belimumab

Twenty-one patients received Belimumab intravenously at standard regimen (10 mg/kg at 0–15–30 days and then every 4 weeks).

Enrolled patients were 2 males and 19 females with a median age of 41 (31–58) years. The disease duration at time of Belimumab start was 10 (2–23) years. The baseline SLEDAI-2K score was 6 (4–10), the anti-dsDNA level was 26 (7–128) UI/ml, and their C3 and C4 level was 72 (52–97) and 9 (6–18) mg/dl, respectively. No significant differences were found between the whole group of patients and the subgroup of patients tested for lymphocyte immunophenotyping (Table 1).

During Belimumab treatment there was a significant improvement in SLEDAI-2K activity index, while no significant change was observed in anti-dsDNA, C3 and C4 levels. Prednisone dosage was progressively reduced, while concomitant immunosuppressive therapies remained unchanged (Table 2).

**TABLE 2:** Comparisons of the clinical and laboratory features of 21 SLE patients at different time points.

	T0 (*n* = 21)	T6 (*n* = 21)	T12 (*n* = 21)	p T0 vs. T6	p T0 vs. T12	p T6 vs. T12
Disease activity						
SLEDAI 2K-score	6 (4–10)	4 (2–6)	4 (2–5)	**0.0007**	**0.0025**	0.9045
Laboratory Parameters						
Serum levels of C3 (mg/dl) (*nv* = 80–160)	72 (52–97)	74 (50–103)	73 (50–101)	0.9404	0.6789	0.6600
Serum levels of C4 (mg/dl) (*nv* = 10–40)	9 (6–18)	11 (3–18)	11 (6–20)	0.3480	0.1394	0.3486
Serum levels of anti-dsDNA (UI/ml) (*nv* < 7)	26 (7–128)	28 (5–228)	28 (5–110)	0.5699	0.2114	0.3396
Treatment						
Dosage of prednisone (mg/day)	9 (5–23)	6 (3.6–12.5)	4 (3–10.5)	**0.0030**	**0.0010**	**0.0468**

Data are expressed as median (10th–90th percentile). SLEDAI-2K score, Systemic Lupus Erythematosus disease Activity Index 2000; C3 and C4: complement factor 3 and 4; Anti-dsDNA, anti-double-stranded DNA autoantibody; nv, normal values. In bold *p* ≤ 0.050.

### Analysis of Changes in B- and T-Cell Compartment During Belimumab Treatment

After treatment with Belimumab, B lymphocytes decreased in patients with SLE, both in percentages (T0, T6 and T12 = 8.1, 3.8 and 3.1 % of CD19 on total lymphocytes) and absolute numbers (T0, T6 and T12 = 82.3, 17.3 and 21.1 cell/μl). In particular, there was a decrease of naïve B cells (T0, T6 and T12 = 45.5, 25.1 and 19.1% of CD19 on total lymphocytes; T0, T6 and T12 = 20.8, 1.5 and 1.4 cell/ul) while percentage switched memory B cells increased (T0, T6 and T12 = 18.4, 41.4 and 48.9% of CD19 on total lymphocytes; T0, T6 and T12 = 13.3, 5.8 and 6.0 cell/ul).

The percentage and the absolute number of unswitched memory and transitional B cells did not change significantly.

Comparing distributions of CD4^+^, CD8^+^, regulatory T cells, and naïve, central memory, effector memory, terminal differentiated effector memory, CD28 negative subsets among CD4^+^ and CD8^+^ T cells, before and after therapy with Belimumab, we did not observe any significant changes over time (Supplementary Table S1). These results confirmed our previous observation in a larger cohort (Regola et al., 2019).

### Analysis of Serum Level Changes of Tumor Necrosis Factor Superfamily Biomarkers During Belimumab Treatment

The following biomarkers belonging to the TNF/TNFR superfamily were tested in serum of the 21 enrolled patients: BAFF, APRIL, sTACI, sBCMA, sCD40L, and TWEAK (Table 3).

**TABLE 3:** Serum Levels Changes of TNF related Biomarkers during belimumab treatment.

	T0 (*n* = 21)	T6 (*n* = 21)	T12 (*n* = 21)	p T0 vs. T6	p T0 vs. T12	p T6 vs T12
BAFF (pg/ml)	444.8 (134.4–1,091.2)	366.5 (201.5–722.7)	334.7 (209.2–589.9)	**0.0547**	**0.0215**	0.8124
APRIL (pg/ml)	2053.3 (1,369.2–3,407.9)	2,085.3 (1,346.8–3,268.5)	2,117.6 (1,368.1–3,862.2)	0.1678	0.4304	0.9273
sTACI (pg/ml)	1,096.5 (397.3–6,599.9)	782.3 (181.7–4,011.3)	777.6 (243.0–4,610.9)	**0.0003**	**0.0006**	**0.0215**
sBCMA (ng/ml)	7,982.7 (6,099.2–12,114.7)	7,954.4 (5,523.5–11,303.0)	7,437.8 (5,771.4–9,319.0)	**0.0319**	**0.0010**	0.1054
sCD40L (pg/ml)	1,817.3 (703.2–10,754.0)	1,243.1 (522.2–4,721.5)	1,326.5 (522.2–5,999.5)	**0.0005**	**0.0136**	0.0759
TWEAK (pg/ml)	1,381.3 (938.9–10,656.5)	1,446.8 (815.5–6,671.6)	1,365.2 (812.0–6,151.8)	**0.0010**	**0.0020**	0.1327

Data are expressed as median (10th–90th percentile). In bold *p* ≤ 0.050. BAFF: B cell activating factor; APRIL: a proliferation-inducing ligand; sTACI: soluble transmembrane activator and calcium-modulator and cyclophilin ligand interactor; sBCMA, soluble B cell maturation antigen; sCD40L: soluble CD40 ligand; TWEAK: TNF-related weak inducer of apoptosis.

Serum levels of BAFF (T0, T6 and T12 = 444.8, 366.5 and 334.7 pg/ml), as well as sTACI (T0, T6 and T12 = 1,096.5, 782.3 and 777.6 pg/ml) significantly decreased over time.

Serum levels of APRIL remained stable over time (T0, T6 and T12 = 2,053.3, 2,085.5 and 2,117.6 pg/ml).

sBCMA serum levels decreased, but significantly only between 6 and 12 months following start of Belimumab treatment (T0, T6 and T12 = 7,982.7, 7,954.4 and 7437.8 ng/ml). On the other hand, changes in sCD40L were observed mostly in the first 6 months of therapy (T0, T6 and T12 = 1,817.3, 1243.1 and 1,326.5 pg/ml).

Serum levels of TWEAK, after an initial increase, significantly decreased one year after initiation of Belimumab treatment (T0, T6 and T12 = 1,381.3, 1,446.8 and 1,365.2 pg/ml).

Serological levels of the TNF/TNFR Superfamily members at different time points in each subject are shown in Figure 1.

### Correlation Between Baseline Number of B- and T-Cell Subsets and Serum Levels of Tumor Necrosis Factor Superfamily Members, and Their Change Over Time

The percentage of CD19 ^+^ cells at baseline showed a correlation with baseline levels of sBCMA (*r* = 0.7, *p* = 0.02) and TWEAK (*r* = 0.86, *p* = 0.002) (Supplementary Table S2).

A significant correlation between the percentage of variation of CD19^+^ and the decrease of sTACI (*r* = 0.7, *p* = 0.02) and TWEAK (*r* = 0.8, *p* = 0.002), respectively, was found at 12 months of follow-up.

No correlation was found between TNF/TNFR superfamily members and the number of T-cell subsets (and their respective variations) (data not shown).

### Determination of Parameters Associated With SLEDAI and Anti-dsDNA Antibody Changes During Belimumab Treatment

Regression analysis confirmed that SLEDAI-2K decreases with duration of Belimumab treatment, with an average reduction of 2.9 (+/− 0.51) at T6 and 3.3 (+/− 0.52) points at T12 (Table 4). In addition, an increase in serum levels of C3 and sTACI was associated with SLEDAI-2K. On average SLEDAI-2K was 0.5 (+/- 0.1) points lower with every 10 mg/dl increase of C3. For sTACI, this association was weaker with SLEDAI-2K reduced by 0.02 (+/− 0.01) points for every 100 pg/ml increase in sTACI (Table 4). When including time after treatment initiation as an interacting factor into the model, the association with C3 remained, however, the association with TACI was not significant anymore (Supplementary Table S3).

**TABLE 4:** Results of robust mixed linear regression model for predictors of SLEDAI-2K. Individual Patient ID was included as random effect in the model. Predictor values were measured at all timepoints.

Dependent: SLEDAI-2K				
Predictors	Estimate	Std. error	*t*. value	*p*
(Intercept)	11.4386	1.1960	9.5644	<0.001
Month 6	**−2.9902**	**0.5108**	**−5.8544**	**<0.001**
Month 12	**−3.3201**	**0.5197**	**−6.3886**	**<0.001**
C3	**−0.0574**	**0.0140**	**−4.1083**	**0.0004**
sTACI	**−0.0002**	**0.0001**	**−2.3424**	**0.0285**

MODEL FIT: AIC = 300.13, BIC = 315.02 Pseudo-R^2^ (fixed effects) = 0.47 Pseudo-R^2^ (total) = 0.63. In bold *p* ≤ 0.050. SLEDAI-2K score, systemic lupus erythematosus disease activity index 2,000; sTACI: soluble transmembrane activator and calcium-modulator and cyclophilin ligand interactor; C3: complement factor 3.

Further mixed linear regression analysis revealed that month into treatment did not significantly change anti-dsDNA serum titers (Table 5). However, an increase of serum C4, sBCMA and/or absolute cell number of CD8^+^ T cells was associated with a decrease, whereas an increase of CD40L and/or number of CD4^+^ T cells was associated with an increase in anti-dsDNA antibody titers (Table 5).

**TABLE 5:** Results of robust mixed linear regression model for predictors of anti-dsDNA antibody titer. Individual Patient ID was included as random effect in the model. Predictor values were measured at all timepoints.

Dependent: anti-dsDNA antibody titer				
Predictors	Estimate	Std. error	*t* value	*P*value
(Intercept)	335.7107	76.5060	4.3880	0.0007
Month 6	37.1296	30.4093	1.2210	0.2369
Month 12	−29.3079	30.7385	−0.9535	0.3528
C4	**−8.0294**	**2.1743**	**−3.6928**	**0.0027**
sBCMA	**−0.0285**	**0.0076**	**−3.7340**	**0.0015**
sCD40L	**0.0308**	**0.0086**	**3.5974**	**0.0018**
#CD4 T cells	**0.4038**	**0.1212**	**3.3317**	**0.0029**
#CD8 T cells	**−0.4150**	**0.1558**	**−2.6639**	**0.0160**

MODEL FIT: AIC = 384.81, BIC = 400.93 Pseudo-*R* ^2^ (fixed effects) = 0.76 Pseudo-*R* ^2^ (total) = 0.87. In bold *p* ≤ 0.050. sBCMA, soluble B cell maturation antigen; sCD40L, soluble CD40 Ligand; C4: complement factor 4; # absolute number of cells.

In a last step, we explored possible predictive models using baseline parameters to estimate percent improvement of SLEDAI-2K after 12 months of therapy. Using stepwise forward inclusion, the minimal model to best describe response to Belimumab after 12 months included BAFF serum level and SLEDAI-2K at baseline. The model predicted more than half of the change in SLEDAI-2K at T12 (*R* ^2^ = 0.61; Adj. *R* ^2^ = 0.56, *p* < 0.01, Table 6). Every increase in serum BAFF of 100 pg/ml at baseline results in a reduction of SLEDAI-2K after one year of treatment of 1.8% (95% CI: 1.4–2.2%) on average. In addition, for every SLEDAI-2K increase of one point at baseline, SLEDAI-2K at T12 into Belimumab treatment decreases about 4.6% (95% CI: 2.6–6.5) (Table 6). Since correlation analysis showed high interdependence of all measured members of the TNF superfamily members, we also determined if BAFF as a predictor of response can be substituted by any of the other determined TNF family members. As shown in Supplementary Table S3 most of the TNF family members will not suffice as predictors for SLEDAI response after 12 months, however, baseline APRIL serum level also showed a significant association, although the overall model fit was slightly weaker than using serum baseline BAFF as predictor (Supplementary Table S4).

**TABLE 6:** Linear regression model to predict percent improvement of SLEDAI-2K after one year, dependent on SLEDAI-2K at baseline and BAFF levels measured at baseline.

Dependent: Percent improvement of SLEDAI2K after one year					
Baseline predictors	Estimate	5%	95%	*t* val.	*p*
(Intercept)	−0.438	−20.491	19.614	−0.038	9.7*e* − 01
BAFF	**0.018**	**0.014**	**0.022**	**8.107**	**3.0*e* − 07**
SLEDAI-2K	**4.574**	**2.646**	**6.501**	**4.128**	**7.0*e* − 04**

MODEL FIT: F (2,17) = 13.27, *p* = <0.01 *R* ^2^ = 0.61 Adj. *R* ^2^ = 0.56. In bold *p* ≤ 0.050. SLEDAI-2K score, systemic Lupus Erythematosus disease activity index 2,000; BAFF: B cell activating factor.

## Discussion

Belimumab, a monoclonal antibody targeting BAFF, has been approved since 2011 as an add-on therapy in adult SLE patients who have an active disease despite standard treatment. Its efficacy and safety were demonstrated in four randomized controlled trials for prolonged use of the drug (Ruiz-Irastorza and Bertsias, 2020). In our cohort, the drug showed its beneficial effects in reducing disease activity over a 12-month-period, as demonstrated by the reduction of the SLEDAI-2K index. However, the variations of anti-dsDNA titer and complement levels were not significant, which was in contrast to previous reports (Furie et al., 2011; Navarra et al., 2011). This difference may be related to the selection of patients who had a mild serological activity at baseline. In general, the main indication for adding Belimumab in our patients was actually to reduce steroid dose. In fact, the well-known effect in reducing cumulative exposure to glucocorticoids was evident as early as 6 months into therapy.

Despite the fact that the post-hoc analysis of trial data showed a greater response in patients with high clinical or serological disease activity (van Vollenhoven et al., 2012), no biomarker has been validated yet for the routine management of patients who are candidate for Belimumab. To address this issue, we considered TNFSF/TNFRSF related factors which had the potential to serve as biomarkers for SLE disease assessment and monitoring of immunomodulatory therapy. In fact, the detection of these factors, which play a role in the pathogenesis of SLE regulating crosstalk between immune cells, could be easily standardized, being measured in peripheral blood in a reproducible way. Among others, circulating levels of BAFF and APRIL, which have an important role in selection, maturation and survival of B cells, are a matter of interest for SLE because their production is enhanced in response to B cell activation through Toll-like receptor (TLR)-9, interferons (IFNs), interleukin (IL)-10 and granulocyte colony-stimulating factor (G-CSF), all involved in SLE pathogenesis (Koyama et al., 2005; Petri et al., 2008; Salazar-Camarena et al., 2016). In addition, an increase in BAFF levels has been described in association with increased disease activity and anti-dsDNA antibodies (Petri et al., 2008). In our study, BAFF progressively reduced over one year of therapy with Belimumab associated with clinical improvement in patients, and a weak correlation with reduction of naïve and transitional B cells, as suggested by the mechanism of action of Belimumab and as reported by us before (Regola et al., 2019). Such correlation was not confirmed in the present study, reinforcing the concept that the main effect of Belimumab on subpopulations could act through the blocking of membrane BAFF, and not of the soluble form (Regola et al., 2019). The post-hoc analysis of phase III randomized clinical trials showed that BAFF levels ≥ 2 ng/ml at enrollment were an independent prognostic factor for an increased risk of moderate and severe lupus flares in patients randomized to receive standard therapy only (Petri et al., 2013). Another analysis from the same study found that patients with BAFF serum levels ≥ 2 ng/ml at baseline had higher response parameters than those with lower BAFF levels, in the Belimumab arm (Petri et al., 2013). In the same way, it was demonstrated that serum BAFF levels ≥ 1.2 ng/ml predicted an increased probability and shorter time to reach response in a cohort of Swedish SLE patients (Parodis et al., 2017). According to this, we demonstrated an association between higher baseline BAFF serum levels and a greater reduction in SLEDAI-2K score after 12 months of therapy, reinforcing the evidence that determination of BAFF levels at the beginning of therapy, together with evaluation of clinical disease activity, could be useful in predicting response to the drug. In our study, BAFF levels decreased during Belimumab therapy, in contrast with what was demonstrated in another report in which BAFF levels increased with time (Parodis et al., 2017). A possible explanation of this observed dissimilarity between studies could be related to the possible different pre-analytic processing of samples which may have caused a modification in the structure of the BAFF molecule, influencing its detection. To better address this aspect, further investigations are necessary to identify if the detected serum BAFF represents only the active form of the molecule or even the inactive form which is complexed with the drug. It has also to be clarified if the proportion of circulating BAFF is representative with the amount that is compartmented in the tissues or expressed on membranes, in order to better identify possible clinical associations.

As an alternative biomarker, although weaker associated with response than BAFF, we showed that baseline serum level of APRIL could also be useful. In the APRIL-SLE clinical trial, BAFF levels above the median at baseline were correlated with an increased risk of British Isles Lupus Assessment Group (BILAG) A or B flare (Gordon et al., 2003) in the placebo group (Isenberg et al., 2015) and patients with high baseline serum values of both BAFF and APRIL showed the greatest effect size. While there are some studies that demonstrated a direct correlation between BAFF and anti-dsDNA serum levels (Stohl et al., 2003; Petri et al., 2008), conflicting results were reported about the possible correlation between APRIL levels and SLE disease activity in terms of activity indices or autoantibody levels (Stohl et al., 2004; Koyama et al., 2005). Furthermore, the reverse trend displayed by APRIL as compared to BAFF confirms the possibility that these two factors could play an opposite role in SLE (Morel et al., 2009).

TACI and BCMA, the common receptor of BAFF and APRIL, were described to be involved in immunoglobulin class switching (He et al., 2010) and in promoting plasma cells survival (O’Connor et al., 2004), respectively. Recent studies demonstrated that the soluble form of these receptors, sTACI and sBCMA, act as decoy receptors with a role in immunomodulatory pathways, being the result of a proteolytic shedding partially dependent on ligand binding and receptor interactions (Meinl et al., 2018). Circulating sTACI, identified as a potential biomarker in autoimmune diseases, is shed from the membrane of activated B cells and plasma cells (Hoffmann et al., 2015). It functions as an immunoregulator, because its decoy function reduces BAFF- and APRIL-mediated survival of different B cell subpopulations (Salazar-Camarena et al., 2020). The reduction of B-cell hyperactivation after therapy with Belimumab could explain the parallel decrease of sTACI, that was demonstrated to be increased in SLE patients in correlation with disease activity (Hoffmann et al., 2015). According with its potential clinical value, we showed that variation in sTACI was related to the improvement of the clinical condition of our patients, as measured by SLEDAI-2K. However, its independent contribution is weak, as demonstrated by our model. Recently, the expression of BCMA on B cells was shown to decrease in active SLE (Salazar-Camarena et al., 2016), however the soluble form, sBCMA, was increased in serum and correlated with disease activity and anti-dsDNA levels (Salazar-Camarena et al., 2020). BCMA was found to be expressed also on the surface of T cells regulating their expansion within the lymph node germinal centers (GC) (Coquery et al., 2015). Recently, it was also demonstrated that the decoy function of circulating sBCMA is only relevant for APRIL and not for BAFF, especially in conditions of over-production, such as in SLE (Coquery et al., 2015). Confirming the relevance of sBCMA as marker of B-cell activation (Laurent et al., 2015; Vincent et al., 2019), we showed an association with autoantibody titers.

The interaction between CD40 on B cells and its ligand (CD40L) which is transiently expressed by T cells and released in soluble form by activated CD4^+^ T cells, is another crucial event that takes place in GC with a role in enhancing humoral response (Basso et al., 2004). It plays a central role in SLE, considering the importance of T-cell dependent humoral immune responses in its pathogenesis (Nagy et al., 2005). Soluble CD40L (sCD40L) in serum or its expression in tissues is upregulated in SLE patients, and often associated with disease severity (Yazdany and Davis, 2004). As shown for sBCMA, its circulating levels revealed an association with anti-dsDNA titers in our cohort, confirming previous findings (Stohl et al., 2003; Petri et al., 2008).

TWEAK is a circulating trimeric molecule which exerts its effect at tissue level. Its relevance in SLE is linked to the constitutive presence in kidneys, with an upregulation during injuries (Schwartz et al., 2006). The lack of association between serum TWEAK levels or their change over time, and clinical or serological parameters of our patients, could be explained by the fact that patients were not enrolled during an acute phase of a nephritis. Its reduction over time in this cohort is unclear and, apparently, without a biological significance. Further evaluations of this tissue-specific parameter could be performed in the future on a specific subset of patients, considering the potential antiproteinuric effects of Belimumab that have emerged from the latest studies (Dooley et al., 2013; Kang et al., 2017).

Some correlations were found between circulating number of B cells and the TNFSF/TNFRSF related factors at baseline. It suggested their inter-relation, but the lack of correlation with specific B-cell subsets may be explained with the presence of other factors which are involved in the maturation and function of B-cells during the disease course. Indeed, the partial evaluation of potentially involved circulating factors, the lack of complementary functional studies, along with the limited number of enrolled patients and the lack of a control group, are limitations of this real-life observational study.

However, our analysis of B- and T-cell compartment modifications during Belimumab therapy sheds a light on the potential usefulness of peripheral B-cell immunophenotyping in SLE patients, confirming our previous results (Regola et al., 2019) and the evidence showed by other researchers in that field (Ramsköld et al., 2019). In particular, it was showed that the long-term longitudinal evaluation of B cells could have important implications in the evaluation of a cellular response to the treatment, that could lead to clinical improvement (Ramsköld et al., 2019). Despite these suggestions, the routinely monitoring of circulating cells could be difficult to be introduced into clinical practice, also on the basis of a lack of evidence in predicting response. In conclusion, only the determination of baseline BAFF serum levels might be useful and feasible to predict the response to therapy, as an add-on biomarker to those already tested in clinical practice.

## Data Availability

The raw data supporting the conclusion of this article will be made available by the authors, without undue reservation.
