# Supplementary material for: Characterization of B- and T-Cell Compartment and B-Cell Related Factors Belonging to the TNF/TNFR Superfamily in Patients With Clinically Active Systemic Lupus Erythematosus: Baseline BAFF Serum Levels Are the Strongest Predictor of Response to Belimumab after Twelve Months of Therapy
Source: Front Pharmacol. 2021 May 21;12:666971. doi: 10.3389/fphar.2021.666971 (PMC8176088; doi:10.3389/fphar.2021.666971)
Supplement: Supplementary file 1 [file DataSheet1.docx]

|  | ***T0 (n=10)*** | ***T6 (n=10)*** | ***T12 (n=10)*** | ***p T0 vs T6*** | ***p T0 vs T12*** | ***p T6 vs T12*** |
| --- | --- | --- | --- | --- | --- | --- |
| CD4+ (% lymphocytes) | 40.2 (28.5-65) | 42.6 (28-55) | 41.0 (33-61) | 0.7896 | 0.2754 | 0.6953 |
| CD4+ (cell/μl) | 381.8 (69-1074 | 325.5 (168-718) | 371.6 (86-600) | 0.1475 | 1.000 | 0.6250 |
| CD4+ naïve (% CD4+) | 36.8 (24-48) | 35.3 (23-50) | 31.2 (22-41) | 1.000 | 0.0244 | 0.0488 |
| CD4+ naïve (cell/μl) | 128.0 (27-327) | 95.0 (45-248) | 108.3 (39-201) | 0.4131 | 0.7695 | 0.5566 |
| CD4+ CM (% CD4+) | 10.4 (3.3-26) | 9.2 (5-23) | 11.0 (3-24) | 0.6377 | 0.4648 | 0.8240 |
| CD4+ CM (cell/μl) | 34.8 (10-99) | 38.0 (8-69) | 42.0 (8-121) | 0.8984 | 0.4316 | 0.5566 |
| CD4+ EM (% CD4+) | 35.8 (27-43) | 39.3 (24-48.5) | 36.8 (22.5-51) | 0.9658 | 0.3203 | 0.3652 |
| CD4+ EM (cell/μl) | 109.1 (25-385) | 95.0 (40-297) | 129.7 (37-235) | 0.2061 | 0.3750 | 0.3750 |
| CD4+ TDEM (% CD4+) | 10.0 (7-34) | 14.7 (10-19) | 18.4 (8-25) | 0.4234 | 0.4496 | 0.6377 |
| CD4+ TDEM (cell/μl) | 31.4 (11-192) | 42.7 (18-77) | 58.6 (25-92) | 0.8311 | 0.6953 | 0.5566 |
| CD4+ CD28- (% CD4+) | 8.9 (5-26) | 7.0 (5-29) | 11.0 (3-23) | 0.5771 | 0.8984 | 0.4648 |
| CD4+ CD28- (cell/μl) | 28.3 (12-82) | 24.3 (12-127) | 25.7 (13-77) | 0.7002 | 0.5566 | 0.5566 |
| CD4+ reg (% CD4+) | 2.0 (1-4) | 3.4 (1-8) | 2.4 (1-3) | 0.3223 | 0.8457 | 0.2023 |
| CD4+ reg (cell/μl) | 8.2 (3-26) | 6.5 (2-37) | 6.6 (3-19) | 1.0000 | 0.6523 | 0.1934 |
| CD8+ (% lymphocytes) | 27.6 (23-43) | 33.5 (19-41) | 33.4 (28-42) | 1.000 | 0.1934 | 1.000 |
| CD8+ (cell/μl) | 356.4 (74-485) | 257.7 (61-422) | 305.5 (83- 454) | 0.1475 | 0.9219 | 0.6953 |
| CD8+ naïve (% CD8+) | 42.8 (10-53) | 37.0 (17-57) | 37.0 (10-68) | 0.5195 | 0.4648 | 0.6250 |
| CD8+ naïve (cell/μl) | 113.1 (32-178) | 73.7 (17-148) | 78.2 (15-211) | 0.2061 | 0.8457 | 0.6250 |
| CD8+ CM (% CD8+) | 1.4 (0.1-3) | 0.6 (0.1-4) | 0.5 (0.2-4) | 0.6886 | 0.8311 | 0.6831 |
| CD8+ CM (cell/μl) | 4.5 (0-9) | 1.0 (0-8) | 1.2 (0-16) | 0.4316 | 0.7344 | 0.4316 |
| CD8+ EM (% CD8+) | 27.3 (15-34) | 24.1 (13.5-37) | 26.3 (9-52) | 0.6377 | 0.3203 | 0.2402 |
| CD8+ EM (cell/μl) | 83.8 (23-120) | 52.6 (8-111) | 69.3 (15-208) | 0.0830 | 0.3223 | 0.0840 |
| CD8+ TDEM (% CD8+) | 33.7 (18.5-61) | 31.2 (24-69.5) | 31.9 (21-48) | 0.4648 | 0.9658 | 0.6250 |
| CD8+ TDEM (cell/μl) | 87.7 (24-294) | 71.7 (16-201) | 99.0 (33-163) | 0.6377 | 0.3750 | 0.6250 |
| CD8+ CD28- (% CD8+) | 29.1 (14-46) | 32.3 (16-55) | 29.2 (16-81) | 0.7002 | 0.4234 | 0.5195 |
| CD8+ CD28- (cell/μl) | 70.9 (22-170) | 54.2 (20-268) | 63.3 (16-324) | 0.6377 | 1.0000 | 0.7695 |
| CD19+ (% lymphocytes) | 8.1 (3-22) | 3.8 (2-7) | 3.1 (1-5) | **0.0117** | **0.0020** | 0.0440 |
| CD19+ (cell/μl) | 82.3 (15-419) | 17.3 (11-46) | 21.1 (6-57) | **0.0137** | **0.0098** | 0.9219 |
| CD19+ SWm (% CD19+) | 18.4 (4-41) | 41.4 (11-58) | 48.9 (12-63) | **0.0059** | **0.0098** | 0.3223 |
| CD19+ SWm (cell/μl) | 13.3 (5-38) | 5.8 (1-13) | 6.0 (1-27) | 0.1123 | 0.4922 | 1.0000 |
| CD19+UNSWm (%CD19+) | 8.1 (1- 14) | 9.7 (3-21) | 5.9 (3-8.5) | 0.1309 | 0.3223 | 0.0743 |
| CD19+UNSWm (cell/μl) | 2.5 (2-11) | 1.9 (1-7) | 1.1 (0-3) | 0.3594 | **0.0124** | 0.1309 |
| CD19+ naïve (% CD19+) | 45.5 (25-87) | 25.1 (10-45) | 19.1 (8-38) | **0.0039** | **0.0039** | 0.2210 |
| CD19+ naïve (cell/μl) | 20.8 (2-23) | 1.5 (1-2) | 1.4 (0-5) | **0.0059** | **0.0108** | 0.3323 |
| CD19+ trans (% CD19+) | 0.4 (0-7) | 0.6 (0-5) | 0.5 (0-6) | 0.8438 | 0.7344 | 0.5703 |
| CD19+ trans (cell/μl) | 0.1 (0-2) | 0 | 0 | 0.1250 | 0.3125 | 0.5827 |

**Supplementary Table 1. Changes in B and T Cell Compartment during Belimumab Treatment.**

Data are expressed as median (10th–90th percentile). CM: central memory; EM: effector memory; TDEM: terminal differentiated effector memory; reg: regulatory; SWm: switched memory; UNSWm: unswitched memory; trans: transitional. In bold p ≤ 0.050.

|  | BAFF  (pg/ml) | APRIL (pg/ml) | sTACI (pg/ml) | sBCMA (ng/ml) | sCD40L (pg/ml) | TWEAK (pg/ml) |
| --- | --- | --- | --- | --- | --- | --- |
| CD19+ (% lymphocytes) | -0.18 (0.6073) | -0.17 (0.6321) | 0.52 (0.1231) | 0.72 (**0.0234**) | 0.47 (0.1663) | 0.86 (**0.0022**) |
| CD19+ (cell/μl) | -0.51 (0.1334) | -0.22 (0.5367) | 0.22 (0.5367) | 0.33 (0.3487) | 0.09 (0.8113) | 0.66 (**0.0438**) |
| CD19+ SWm (% CD19+) | 0.32 (0.3679) | -0.03 (0.9460) | 0.06 (0.8651) | -0.10 (0.7850) | 0.17 (0.6321) | -0.50 (0.1440) |
| CD19+UNSWm (%CD19+) | 0.45 (0.1912) | 0.05 (0.8916) | -0.07 (0.8382) | -0.11 (0.7589) | 0.09 (0.8113) | -0.54 (0.1139) |
| CD19+ naïve (% CD19+) | -0.34 (0.3304) | 0.01 (1) | 0.13 (0.7072) | 0.10 (0.7850) | 0.11 (0.7589) | 0.59 (0.07) |
| CD19+ trans (% CD19+) | -0.63 (0.06) | 0.16 (0.6567) | -0.19 (0.5837) | 0.13 (0.70) | -0.30 (0.3869) | -0.03 (0.9184) |

**Supplementary Table 2. Correlations between B- cell percentage number and TNF Superfamily Members at baseline.** Data are expressed as r (p). SWm: switched memory; UNSWm: unswitched memory; trans: transitional. In bold p ≤ 0.050.

| Dependent: SLEDAI2K | |  |  |  |
| --- | --- | --- | --- | --- |
| Predictors | Estimate | Std..Error | t.value | p-value |
| (Intercept) | 11.8886 | 1.6028 | 7.4176 | 0.0000 |
| Month 6 | -3.1117 | 2.0403 | -1.5251 | 0.1359 |
| **Month 12** | **-4.6606** | **2.2314** | **-2.0886** | **0.0436** |
| **C3** | **-0.0626** | **0.0196** | **-3.1940** | **0.0024** |
| sTACI | -0.0002 | 0.0001 | -1.8959 | 0.0638 |
| Month 6:C3 | 0.0010 | 0.0249 | 0.0384 | 0.9696 |
| Month 12:C3 | 0.0174 | 0.0275 | 0.6330 | 0.5305 |
| Month 6:sTACI | 0.0000 | 0.0001 | 0.1072 | 0.9152 |
| Month 12:sTACI | 0.0000 | 0.0001 | 0.0567 | 0.9551 |

**MODEL FIT:**

AIC = 350.23, BIC = 373.63

Pseudo-R² (fixed effects) = 0.46

Pseudo-R² (total) = 0.60

**Supplementary Table 3.**

Results of robust mixed linear regression model for predictors of SLEDAI-2K. Individual Patient ID was included as random effect in the model. Predictor values were determined at each time point. In bold p ≤ 0.050. SLEDAI-2K score, Systemic Lupus Erythematosus Disease Activity Index 2000; C3: complement factor 3; sTACI: soluble transmembrane activator and calcium-modulator and cytophilin ligand interactor.

| Predicted: Percent improvement of SLEDAI2K after one year | | | | | |
| --- | --- | --- | --- | --- | --- |
| **Predictors** | **Est.** | **5%** | **95%** | **t val.** | **p** |
| (Intercept) | 2.6204 | -2.5e+01 | 29.918 | 0.17 | 0.8694 |
| sTACI | 0.0021 | -8.4e-04 | 0.005 | 1.24 | 0.2304 |
| **SLEDAI2K** | **4.9741** | **2.4e+00** | **7.570** | **3.33** | **0.0039** |

**MODEL FIT:**

F(2,17) = 5.25, p = 0.02

R² = 0.38

Adj. R² = 0.31

| Predicted: Percent improvement of SLEDAI2K after one year | | | | | |
| --- | --- | --- | --- | --- | --- |
| **Predictors** | **Est.** | **5%** | **95%** | **t val.** | **p** |
| (Intercept) | -4.0377 | -25.6829 | 17.6074 | -0.32 | 0.74951 |
| **APRIL** | **0.0052** | **0.0023** | **0.0081** | **3.09** | **0.00659** |
| **SLEDAI2K** | **4.5618** | **2.6509** | **6.4728** | **4.15** | **0.00067** |

**MODEL FIT:**

F(2,17) = 11.51, p = 0.00

R² = 0.58

Adj. R² = 0.53

| Predicted: Percent improvement of SLEDAI2K after one year | | | | | |
| --- | --- | --- | --- | --- | --- |
| **Predictors** | **Est.** | **5%** | **95%** | **t val.** | **p** |
| (Intercept) | 6.5585 | -1.7e+01 | 29.6605 | 0.49 | 0.6277 |
| sCD40L | 0.0016 | -9.1e-05 | 0.0032 | 1.64 | 0.1184 |
| **SLEDAI2K** | **4.3848** | **2.3e+00** | **6.5091** | **3.59** | **0.0023** |

**MODEL FIT:**

F(2,17) = 4.93, p = 0.02

R² = 0.37

Adj. R² = 0.29

| Predicted: Percent improvement of SLEDAI2K after one year | | | | | |
| --- | --- | --- | --- | --- | --- |
| **Predictors** | **Est.** | **5%** | **95%** | **t val.** | **p** |
| (Intercept) | 3.3170 | -23.4950 | 30.1289 | 0.22 | 0.8322 |
| TWEAK | 0.0012 | -0.0011 | 0.0034 | 0.90 | 0.3784 |
| **SLEDAI2K** | **4.9360** | **2.4199** | **7.4521** | **3.41** | **0.0033** |

**MODEL FIT:**

F(2,17) = 5.46, p = 0.01

R² = 0.39

Adj. R² = 0.32

| Predicted: Percent improvement of SLEDAI2K after one year | | | | | |
| --- | --- | --- | --- | --- | --- |
| **Predictors** | **Est.** | **5%** | **95%** | **t val.** | **p** |
| (Intercept) | -6.5234 | -44.7789 | 31.7321 | -0.3 | 0.7703 |
| sBCMA | 0.0018 | -0.0012 | 0.0048 | 1.0 | 0.3179 |
| **SLEDAI2K** | **4.6949** | **2.2206** | **7.1692** | **3.3** | **0.0042** |

**MODEL FIT:**

F(2,17) = 5.46, p = 0.01

R² = 0.39

Adj. R² = 0.32

**Supplementary Table 4.** Linear regression model to predict percent improvement of SLEDAI-2K after one year, dependent on SLEDAI-2K and one additional predictor, both determined at baseline. In bold p ≤ 0.050. SLEDAI-2K score, Systemic Lupus Erythematosus Disease Activity Index 2000; APRIL: a proliferation-inducing ligand; sTACI: soluble transmembrane activator and calcium-modulator and cyclophilin ligand interactor; sBCMA, soluble B cell maturation antigen; sCD40L: soluble CD40 ligand; TWEAK: TNF-related weak inducer of apoptosis.
